# Multifaceted response of microglia in the cuprizone model of demyelination

**DOI:** 10.1371/journal.pone.0355981

**Published:** 2026-08-26

**Authors:** Ashley Shanahan, Gwenyth Eichfeld, Levi Todd, Anzela Niraula

**Affiliations:** 1 Neuroscience Program, Department of Psychological and Brain Sciences, Colgate University, Hamilton, New York, United States of America; 2 Department of Ophthalmology and Visual Sciences, SUNY Upstate Medical University, Syracuse, New York, United States of America; University of Rijeka Faculty of Health Studies: Sveuciliste u Rijeci Fakultet zdravstvenih studija, CROATIA

## Abstract

The cuprizone diet paradigm, which involves selective depletion of oligodendrocytes and subsequent myelin loss, provides an excellent model to study the neuroimmune response to demyelination and remyelination. Microglia, the resident immune cells of the central nervous system, mount a robust response to cuprizone administration. However, the specific triggers and temporal trajectory of microglia activation are not well-defined. Moreover, it is unclear the extent to which microglia respond to cuprizone directly rather than to cuprizone-induced demyelination. In this study, we characterized the temporal dynamics of microglial activation in the corpus callosum during the early and peak phases of demyelination and during early remyelination following cuprizone withdrawal. Microglia number and area showed a dramatic increase by 1 week (early phase) and persisted throughout 4 weeks (peak phase), with partial recovery during early remyelination. The early demyelination phase was marked by morphological remodeling of microglia and engulfment of degraded myelin. This timepoint correlated with increased expression of *Mertk and Apoe,* and a concurrent decrease in the proinflammatory response genes, *Tnf* and *Ifnb1*. These findings indicate that the early neuroimmune response to cuprizone-induced demyelination is marked by an augmented phagocytic response accompanied by downregulation in inflammatory signaling. In the retina, which is devoid of oligodendrocytes or myelin, microglia did not display morphological alterations and instead downregulated CD68 expression in response to cuprizone administration. Our novel findings reveal that in addition to the microglial response to cuprizone-induced demyelination, cuprizone exerts direct effects on microglia even in absence of *in vivo* demyelination.

## Introduction

Demyelinating diseases result in a loss of myelin and disruptions in axonal conduction, leading to axonal degeneration and motor impairments [1]. Example, Multiple sclerosis (MS) is a demyelinating disease of the central nervous system that affects over 2 million people worldwide [1]. Remyelination following myelin loss in conditions such as MS is associated with reduced disability and functional recovery [2]. The cuprizone diet serves as a model for examining the mechanisms underlying demyelination and remyelination [3,4]. Cuprizone is a toxin administered in the rodent diet to induce oligodendrocyte death and demyelination, particularly in heavily myelinated areas such as the corpus callosum. Cessation of cuprizone leads to subsequent remyelination, and clearance of myelin debris is believed to be crucial for remyelination and recovery [5,6]. Cuprizone administration triggers activation of microglia, the resident immune cells of the central nervous system [7]. The role of microglia in cuprizone-induced demyelination is complex and dependent on treatment duration [8,9]. As oligodendrocytes undergo cell death, microglia clear lipid and cholesterol-rich lytic carcasses [3,10]. Microglia also clear myelin debris, and secrete trophic factors to promote oligodendrocyte growth and survival [5,11,12]. In addition, microglia produce inflammatory cytokines and mediators of oxidative stress during demyelination [10,13]. Perplexingly, microglia contribute to both cuprizone-induced demyelination and remyelination following cuprizone withdrawal [14–16]. Marzan et al. (2021) showed microglia depletion during cuprizone administration abolished the demyelination and oligodendrocyte loss [15]. In contrast, genetic deletion of *Mertk*, a gene highly expressed in microglia and associated with the clearance of apoptotic cells [17], delayed remyelination following cuprizone withdrawal [6]. These reports indicate a multifaceted role for microglia during demyelination and remyelination.

Recent transcriptomics studies have shed light on the molecular changes that underlie microglial response to cuprizone-induced demyelination, however, these studies have focused on the microglia phenotype weeks into cuprizone administration. One study identified a microglial subpopulation enriched for markers associated with phagocytosis, as well as inflammatory markers similar to those observed in a lipopolysaccharide model [10]. In another study, microglia displayed a disease-associated microglia state [18], along with a heterogeneous profile marked by lysosomal stress and interferon signaling response [13]. These studies examined microglial changes following five weeks of cuprizone administration. Given the dynamic nature of microglia and the transitional shifts microglia undergo over this period, we sought to examine the early microglial response to cuprizone administration. A better understanding of the early microglial response is important for identifying the shifts that determine the adaptive and maladaptive contributions of microglia to demyelination.

Notably, despite the widespread use of the cuprizone diet as a model for demyelination, the direct effects of cuprizone on microglia are not well-described [3,4]. The microglial response to cuprizone administration is largely attributed to oligodendrocyte death and myelin disintegration, however, the extent to which cuprizone directly impacts microglia functions remains unclear [3,19].

This study profiles the microglial response to cuprizone administration at various timepoints: early demyelination (1 and 2 week), peak demyelination (4 week) and early remyelination (4 weeks of demyelination followed by a 1-week recovery). Specifically, we assessed microglia morphology and phagocytic profile at each timepoint of cuprizone administration. We report that microglia adopt a phagocytic state and engulf degraded myelin within a week, and this correlates with attenuated inflammatory signaling in the microenvironment. Lastly, using *in vitro* and *in vivo* experiments, we show that cuprizone exerts direct effects on microglia independent of oligodendrocyte death or myelin loss.

## Materials and methods

### Mice

Female *C57BL/6* wildtype mice (8−10 weeks old) were purchased from the Jackson Laboratories (Bar Harbor, ME). Mice were group-housed on a 12-hour light-dark cycle at 22−25°C temperatures with *ad libitum* access to food and water. All protocols and experiments were carried out in accordance with policies and procedures described in the Guide for the Care and Use of Laboratory Animals of the National Institutes of Health and were approved by the Animal Care and Use Committee at Colgate University (Approval #2324−10).

### Cuprizone administration

Cuprizone diet (Teklad^TM^ Cat #TD.140804) was purchased from Envigo LLC (Indianapolis, IN). Mice were placed on the 0.2% cuprizone diet for 1, 2, or 4 weeks to induce demyelination. A recovery group was administered the cuprizone diet for 4 weeks, followed by standard chow without cuprizone for 1 week. Control animals were placed on the standard chow without cuprizone for 4 weeks (Teklad^TM^ Cat #T.2018.15). Fresh diet was provided every two days and body weight and food weight measurements were taken at the same time.

### Brain sample preparation for immunohistochemistry

At the end of the experiment, mice were euthanized with 5% isoflurane followed by transcardial perfusion with phosphate buffered saline (PBS) and 4% paraformaldehyde (PFA). Brains were extracted and post-fixed in 4% PFA for 24 hours at 4°C, then transferred to 25% sucrose for 48 hours at 4°C. Brains were frozen in isopentane and dry ice, and stored at −80°C until sectioning.

For retina experiments, a separate group of mice was placed on the 0.2% cuprizone diet or standard chow without cuprizone. Following isoflurane anesthesia, mice were transcardially perfused with PBS, cornea removed, and eye globes fixed in 4% PFA for two hours.

### Immunofluorescence labeling

Brains were cryo-sectioned on the Leica CM3050S cryostat and 30-micron free-floating sections were collected in freezing solution and stored at −20°C. For immunolabeling, sections from 1.09 mm to 0.13 mm Bregma (Paxinos and Franklin, 2019) were rinsed in 1X PBS and blocked with 5% normal donkey serum for 1 hour at room temperature. Sections were then incubated overnight at 4°C with the following primary antibodies: mouse anti-CC1, 1:500, Cat #OP80100UG, Millipore Sigma; rabbit anti-BCAS1, 1:1000, Cat #445003, Synaptic Systems; chicken anti-MBP, 1:1000, Cat #2313550, Aves Labs; rabbit anti-Iba-1, 1:1000, Cat #019−19741, Wako; goat anti-Iba-1, 1:1000, Cat #NB100−1028, Novus Biologicals; rat anti-CD68, 1:500, Cat #50-112-9259, Invitrogen. Sections were washed with 1X PBS three times and incubated for one hour at room temperature with the following secondary donkey antibodies at 1:1000: anti-mouse Alexa Fluor^TM^ 594, anti-chicken Alexa Fluor^TM^ 594, anti-rabbit Alexa Fluor^TM^ 488, anti-goat Alexa Fluor^TM^ 594, and anti-rat Alexa Fluor^TM^ 594. Next, sections were stained with DAPI (Cat #EN62248, Thermo Scientific, 1:3000) and washed with PBS. Sections were mounted in Fluoromount-G Mounting Medium (Sigma Cat# 00-4958-02) on Superfrost microscope slides, coverslipped, and stored at 4°C until imaging.

For the retina labeling, PFA-fixed retinal wholemounts were incubated in primary antibodies (Iba-1 and CD68) overnight at room temperature. Wholemounts were then washed with 1X PBS and incubated in secondary antibodies for four hours at room temperature. After washing twice with 1X PBS, retinas were cut and mounted in Fluoromount-G Mounting Medium.

### Epifluorescence imaging and analysis

Epifluorescence imaging was performed on the Keyence BZ-X810 microscope. Sections were imaged at 20X magnification for CC-1, BCAS-1, MBP, and Iba-1 signals. A region of interest (ROI) was created on the medial corpus callosum region or the retina, and images were analyzed using ImageJ Fiji (National Institutes of Health, Maryland, USA). Briefly, images were converted to 8-bit and thresholding settings were used to measure signal percent areas for myelin basic protein (MBP) and Iba-1. MBP percent area was calculated relative to the control animals. Cell counting for mature oligodendrocytes, early myelinating oligodendrocytes, and microglia were performed manually using the Cell Counter function on the CC-1, BCAS-1, and Iba-1 images, respectively. All imaging and analysis were conducted in a blinded manner.

### Confocal imaging and analysis

For morphological and phenotypic assessments of microglia, z-stack images were collected at 40X using the Zeiss LSM 710 confocal microscope. Iba-1 and CD68 images were analyzed for CD68 punctate expression in the Iba-1 positive cells. The number of CD68 punctate per Iba-1 cell and CD68 size were measured using the Cell Counter plugin and Analyze Particles plugin on ImageJ. Microglia engulfment of myelin was assessed using confocal z-stacks containing the Iba-1 channel and MBP (or dMBP) channel. Individual microglia were isolated using an ROI on ImageJ Fiji, and the Threshold function was applied to generate masks of the Iba-1 channel and MBP (or dMBP) channel. The Image Calculator function was used to quantify the volume of overlapping signal objects between the two channels. The overlapping signal is represented as internalized MBP (or dMBP) volume per microglia. Approximately ten microglia were analyzed at random per animal.

For microglia Sholl analysis, the Iba-1 z-stack images were imported into Synaptic Neurite Tracer under the Neuroanatomy plugin on ImageJ. Each microglia cell was traced along the z-stack and Sholl analysis was performed using the Sholl plugin. Microglia branches were analyzed using the Analyze Skeleton feature. These morphological assessments were performed on corpus callosum microglia and ganglion cell layer microglia in the retina.

### Tissue collection and gene expression analysis

In a separate experiment, eight-week old female *C57BL/6* wildtype mice were placed on the 0.2% cuprizone diet or standard chow without cuprizone for a week. Mice were deeply anesthetized with isoflurane and perfused with PBS prior to brain extraction. Next, brains were dissociated in PBS using the Potter-Elehjem tissue homogenizer (Cat #8355-10, Ace Glass) and centrifuged at 400g for 10 minutes at 4°C to obtain a cell pellet. The cells were resuspended in 33% isotonic Percoll solution (Cat #45-001-748, Cytiva) and centrifuged at 400 g for 15 minutes at room temperature. The myelin layer and supernatant were discarded. The pellet was resuspended in PBS, debris filtered out using a cell strainer, and cells centrifuged to obtain a final pellet for subsequent RNA isolation.

RNA isolation was performed following manufacturer’s instructions using the Qiagen RNeasy Micro Kit (Cat #74004, Qiagen). RNA was quantified using the Nanodrop (Thermo Fisher Scientific). The High-Capacity cDNA Reverse Transcription Kit was used for cDNA synthesis (Cat #4368814, Applied Biosystems), and real-time quantitative PCR was performed on the QuantStudio™ 5 system (Thermo Fisher Scientific) using the SYBR Green Mix. Custom-designed primers were purchased from Integrated DNA Technologies. Gene expression was represented as the fold change determined using the ΔΔCt method. 18s was used as the housekeeping gene.

### In vitro experiment for gene expression analysis

BV-2 cells were a kind gift from Dr. William Kerr at SUNY Upstate University. BV-2 cells were plated onto a 24-well plate (50,000 cells/plate) in DMEM media with 5% fetal bovine serum and 1% penicillin streptomycin. Cells were incubated at 37°C and 5% CO_2_ for 24 hours. Upon 90% cell confluence, the media was replaced with serum-free media and incubated overnight. Next day, cuprizone (Cat #C9012-25G, Millipore Sigma) was prepared in a fresh solution as follows: stock solution of cuprizone was prepared in 50% ethanol/water solution, incubated in 60°C water bath, and repeatedly vortexed until cuprizone was fully dissolved. The stock solution was added to the cell media to achieve a final concentration of 25 µM containing 0.125% ethanol. Vehicle wells were treated with media containing 0.125% ethanol. Following a 24-hour treatment, media was removed and cells were lysed. RNA extraction and the subsequent process for gene expression analysis was performed as described in the section above.

### Phagocytosis assay

BV-2 cells were plated on coverslips and prepared as described above. Following overnight incubation in serum-free media, the cells were treated with fluorescent latex beads (L-3030, Sigma-Aldrich) in the presence of 25 μM cuprizone or vehicle for 24 hours. Next, the cells were washed in PBS and fixed in 4% PFA for 30 minutes at room temperature, followed by DAPI staining. Cell coverslips were removed and mounted on slides, and stored at 4°C until imaging. Confocal z-stacks were obtained at 40x magnification using a Zeiss LSM 710. Images were obtained from a consistent location on the coverslip for each sample. To quantify bead engulfment, the number of beads per cell was counted for each condition.

### Statistical analysis

Statistical analyses were performed using GraphPad Prism version 10.2.2 and are presented as mean ± SEM. Group comparisons were conducted using a two-tailed unpaired t-test or a one-way ANOVA with Sidak’s post hoc test for multiple comparisons. Sholl Analysis data were analyzed using two-way ANOVA (Fig 2H). Relationships between mRNA levels were assessed using single linear regression (Fig 4G&H). Differences with a p < 0.05 were considered statistically significant.

## Results

### Cuprizone leads to a rapid decline in oligodendrocyte numbers and myelin in the medial corpus callosum

**Fig 1:**
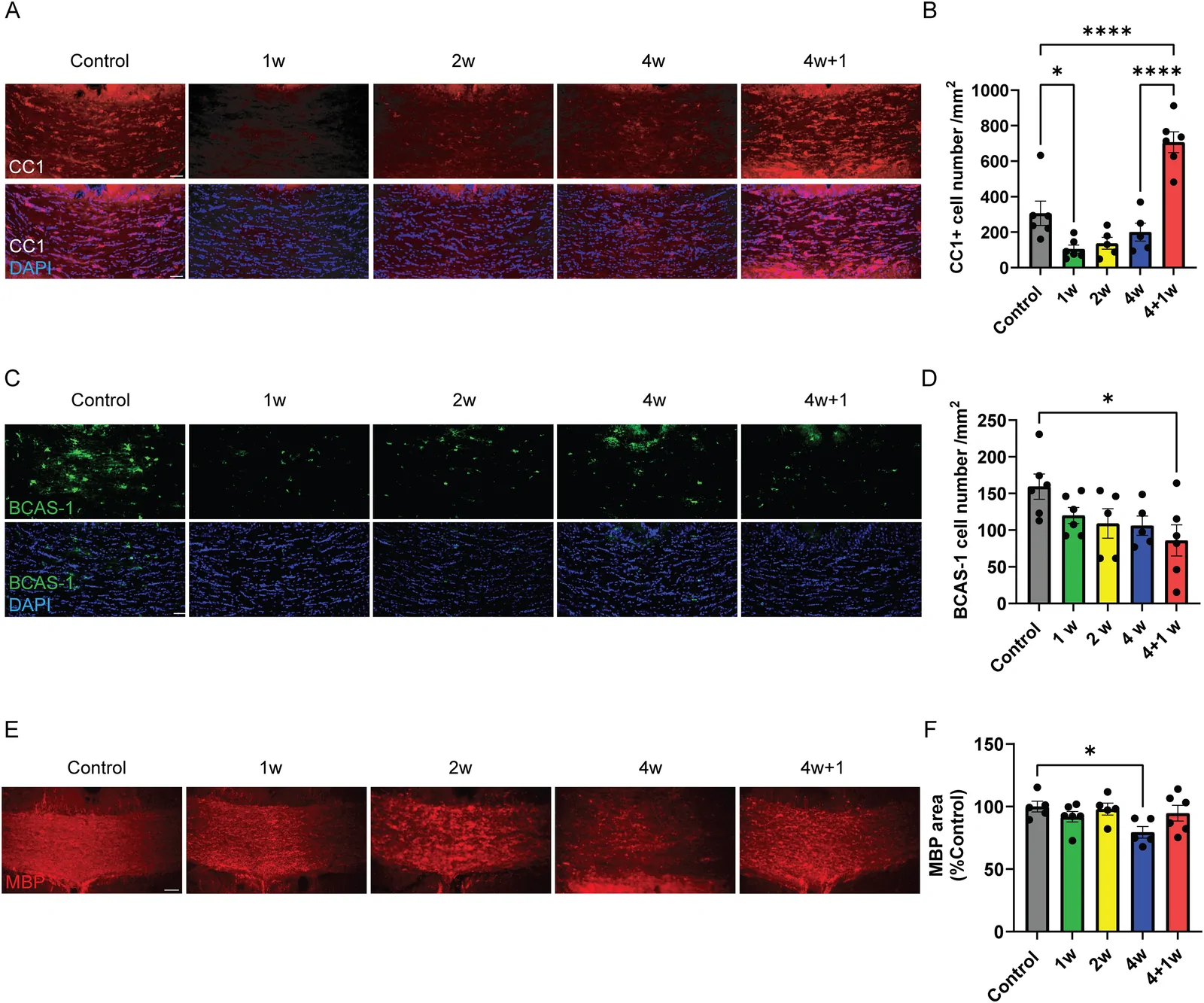
Cuprizone leads to a rapid decline in oligodendrocyte numbers and myelin in the medial corpus callosum (A) Representative images and (B) quantitative analysis of CC1 + cell number in the medial corpus callosum from mice administered with a control diet, 1 week cuprizone (1w), 2 week cuprizone (2w), 4 week cuprizone (4w), or 4 week cuprizone + 1 week withdrawal (4 + 1w). (C) Representative images and (D) quantitative analysis of BCAS-1 + cell number in the medial corpus callosum. (E) Representative images (F) and quantitative analysis of myelin basic protein (MBP) area in medial corpus callosum relative to control. Data presented as mean±SEM and were analyzed using one-way ANOVA followed by Sidak’s post hoc test for multiple comparisons, n = 5-6, *p < 0.05, **p < 0.01, ***p < 0.001, ****p < 0.0001. Scale bar = 50 µm.

To examine the time-course of oligodendrocyte decline and repopulation, we placed mice on a 0.2% cuprizone diet for 1, 2, or 4-weeks of cuprizone administration followed by 1 week on the standard chow diet to examine the subsequent remyelination. The number of CC-1 positive cells in the medial corpus callosum decreased within the first week of cuprizone feeding indicating rapid decline in the oligodendrocyte population ([F(4, 23) = 24.20, p < 0.0001]; p = 0.032 for 1 w v. Control; Fig 1A,B). Upon cessation of the cuprizone diet, there was a rapid repopulation of oligodendrocytes as indicated by a significant difference between the 4 + 1 w group and the 4 w group (p < 0.0001; Fig 1B). Strikingly, oligodendrocyte numbers in the 4 + 1 w group were also significantly greater than in the Control group (p < 0.0001; Fig 1A,B), revealing an overshoot in the mature oligodendrocyte population during early recovery from demyelination.

The number of CC-1 cells in the 2 w or 4 w group did not significantly differ from the Control group (Fig 1A,B). To examine whether the CC-1 population is partially replenished by precursor cells, we conducted immunofluorescence labeling for BCAS-1 cells, which represent a pool of early myelinating oligodendrocytes capable of differentiating into mature oligodendrocytes [20]. The BCAS-1 cell number showed a steady decline across the experimental groups ([F(4, 23) = 2.682, p = 0.057]; Fig 1C,D) although the cuprizone-treated groups were not significantly different compared with the Control. However, the decrease in the number of BCAS-1 cells in the 4 + 1 w group was statistically significant compared with the Control (p = 0.017; Fig 1C,D), indicating that the early myelinating oligodendrocyte numbers decline during early remyelination and that these cells may differentiate and contribute to the mature oligodendrocyte pool.

To examine the corresponding changes in myelin over the course of demyelination and remyelination, we next analyzed myelin basic protein (MBP) levels in the medial corpus callosum. There was a trend towards a significant decrease in MBP area with cuprizone feeding ([F(4, 22) = 2.429, p = 0.0782]; Fig 1E,F). Specifically, MBP levels were significantly reduced at 4 weeks of cuprizone administration (p = 0.0312; Fig 1E,F). This finding is consistent with that of previous studies showing that a noticeable decline in MBP levels does not occur until approximately four weeks of cuprizone administration [3,8]. Nonetheless, the MBP labeling in the 1 w group appeared significantly altered compared with the Control group. The MBP signal displayed a discontinuous and clumpy appearance within a week of cuprizone administration, and this dysmorphic appearance of MBP was more prominent in the 2w and the 4w groups. These findings suggest an early decline in myelin integrity followed by a progressive loss of myelin in the corpus callosum over prolonged cuprizone feeding.

### Cuprizone administration triggered rapid microglia activation, which persisted during early remyelination

**Fig 2:**
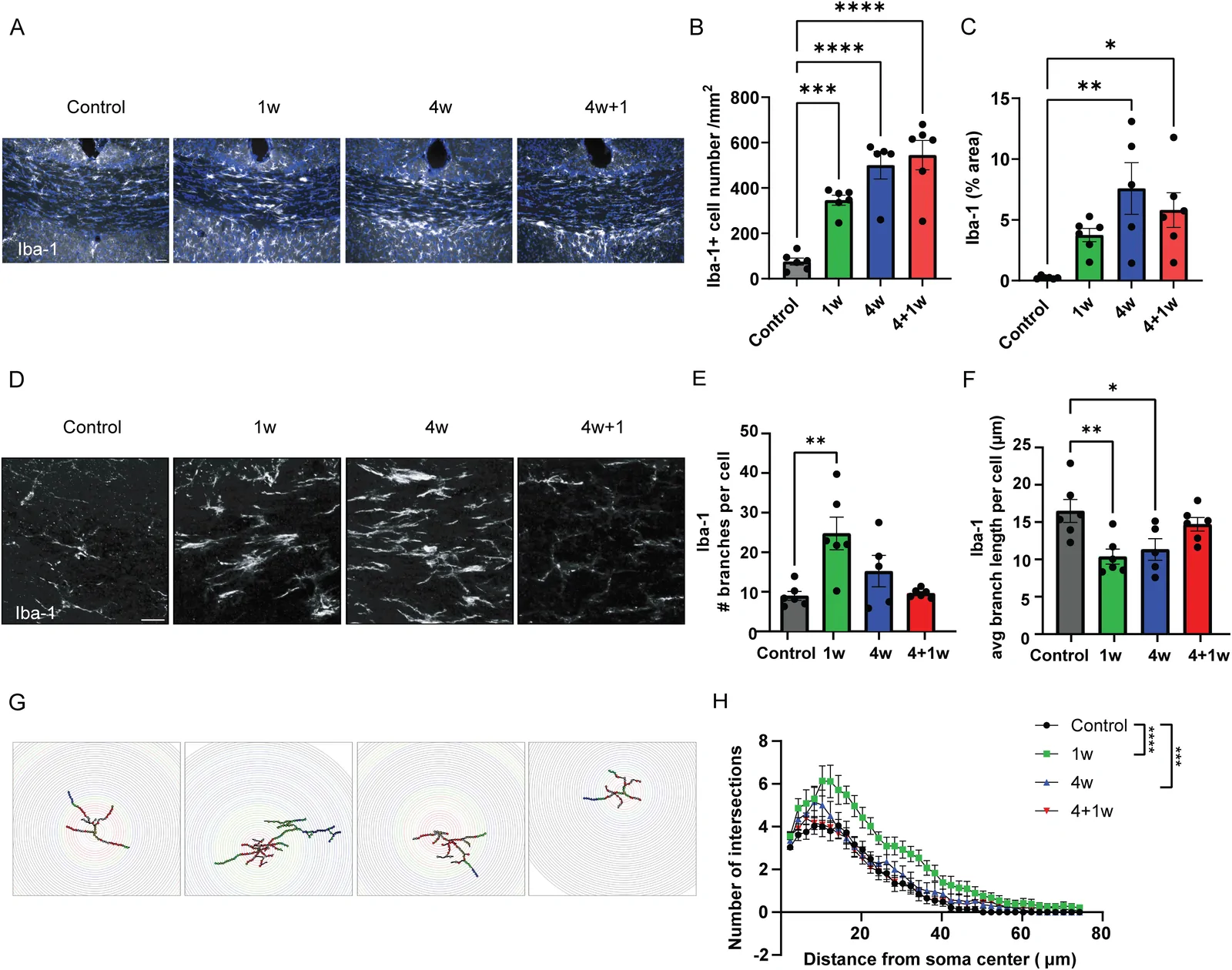
Cuprizone administration triggered rapid microglia activation, which persisted during early remyelination. (A) Representative images and quantification of (B) Iba-1 + cell number and (C) Iba-1 + percent area in medial corpus callosum from mice administered with a control diet, 1 week cuprizone (1w), 4 week cuprizone (4w), and 4 week cuprizone + 1 week withdrawal (4 + 1w). (D) Representative images depicting maximum projection intensity of microglia from confocal z-stacks of Iba-1 positive cells. Skeletal analysis and quantification of (E) microglial branches and (F) average branch length per microglia. (G) Representative reconstructions and (H) quantification of microglia based on Sholl analysis. Data presented as mean±SEM and were analyzed using one-way ANOVA (Fig H analyzed using two-way ANOVA) followed by Sidak’s post hoc test for multiple comparisons, n = 5-6, *p < 0.05, **p < 0.01, ***p < 0.001, ****p < 0.0001. Scale bar = (A) 50 µm (D) 20 µm.

One of the hallmarks of the cuprizone model is the recruitment of activated microglia [7,8,11]. Several studies have reported on the molecular mechanisms governing microglial response at 4−5 weeks following cuprizone administration, which coincides with extensive demyelination. Given our findings that cuprizone leads to demyelination in as little as 1 week we examined the microglial response at this early phase (1 w), peak demyelination phase (4 w), and during early remyelination (4 + 1 w). Immunofluorescence labeling of Iba-1 positive cells revealed a significant increase in microglia number with cuprizone administration ([F(3,19) = 22.82, p < 0.0001]; Fig 2A,B). Specifically, the number of Iba-1 positive cells increased within one week of cuprizone administration (p = 0.0009; Fig 2B), a trend that continued over the weeks and was maintained in the 4 + 1 w group (p < 0.0001 for 4 w v. Control, p < 0.0001 for 4 + 1 w v. Control; Fig 2A,B). Corresponding with the increase in Iba-1 cell number, the Iba-1 percent area was greater with cuprizone administration ([F(3,19)=6.656, p = 0.003]; Fig 2A,C). Specifically, the 4 w cuprizone group was significantly different compared with the Control (p = 0.0015; Fig 2C). The number and percent area covered by Iba-1 + cells remained elevated in the recovery period following 4 w cuprizone compared with the Control (4 + 1 w v. Control, p = 0.011; Fig 2C). Taken together, microglial response to cuprizone administration was characterized by an expansion in cell population and hypertrophy, and this response was maintained during early remyelination.

To quantify the structural changes in microglia during demyelination and remyelination, we conducted a Skeletal Analysis of the Iba-1 confocal z-stack images. The number of branches ([F(3,19) = 6.999, p = 0.0023]; Fig 2D,E) and the average branch length ([F(3,19) = 5.425, p = 0.0072]; Fig 2D,F) were significantly altered with cuprizone administration. The Iba-1 positive cells in the 1 w group showed increased branching (p = 0.002; Fig 2E), but the branches were shorter on average (p = 0.006; Fig 2F) compared with the Control. The shorter branch length was maintained in the 4 w group (p = 0.029; Fig 2F). Notably, the 4 + 1 w group did not differ from the Control group in branch number or branch length (Fig 2E,F). To further characterize the morphological remodeling in microglia, we conducted Sholl Analysis on the confocal z-stack images of Iba-1 positive cells in the corpus callosum. Sholl analysis revealed a significant difference in the number of intersections of Iba-1 processes with cuprizone feeding (Treatment [F(3.703) = 91.78, p < 0.0001]; Distance from Soma [F(36, 703) = 116.8, p < 0.0001]; Treatment x Distance [F(108,703) = 1.397, p = 0.008]; Fig 2G,H). Specifically, there was an increase in the number of intersections of Iba-1 processes in the 1 w group and 4 w group compared with the Control (p < 0.0001 for 1 w v. Control, p = 0.0005 for 4 w v. Control; Fig 2G,H). This indicates that cuprizone triggered an increase in microglial ramifications. Taken together, the rapid microglia activation within a week of cuprizone administration is marked by significant morphological remodeling that persists during peak demyelination and partially recovers during early remyelination.

### The early microglial response to cuprizone administration was marked by a phagocytic profile and engulfment of degraded myelin

**Fig 3:**
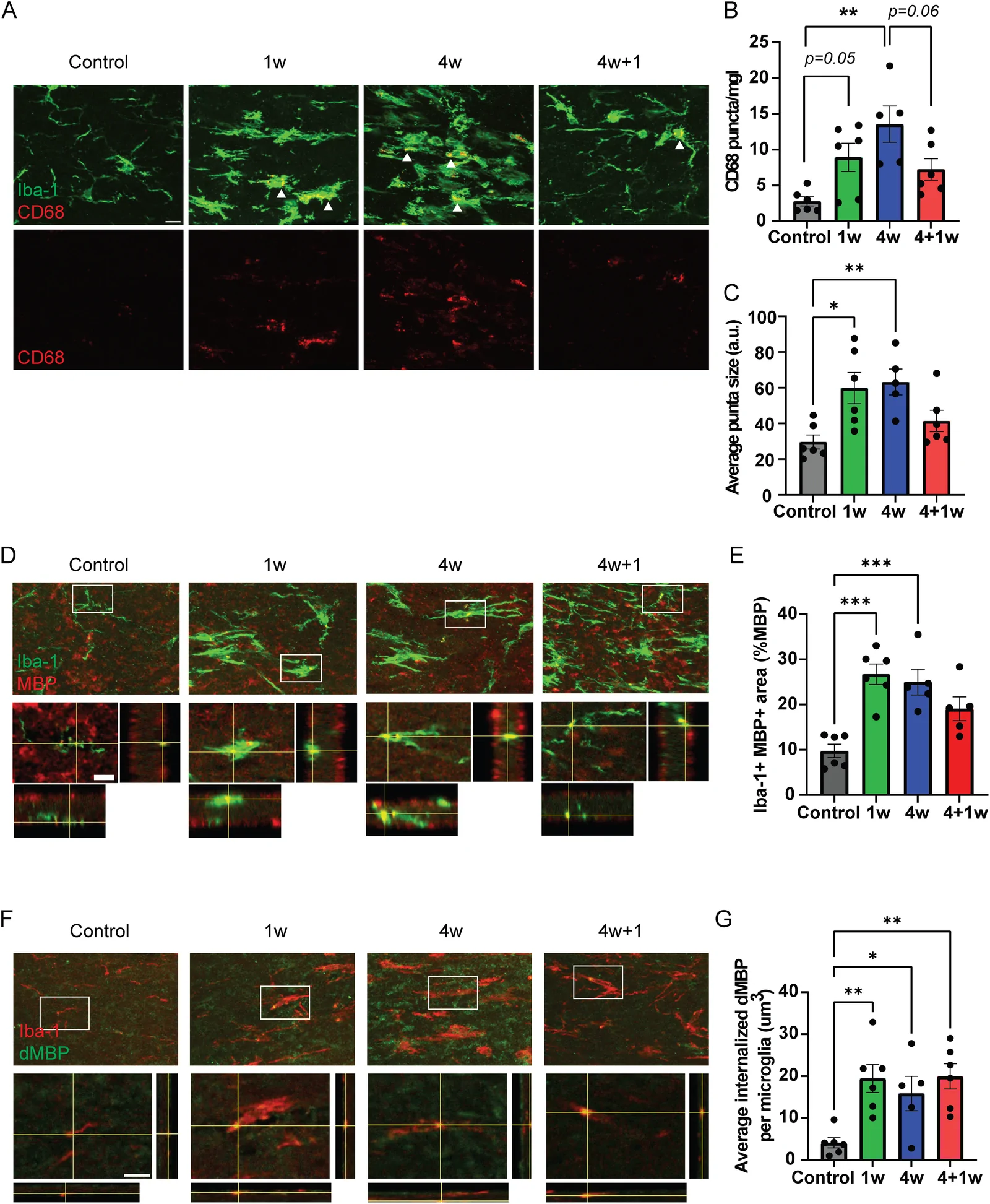
The early microglial response to cuprizone administration was marked by a phagocytic profile and engulfment of degraded myelin. (A) Representative confocal z-stack images of Iba-1 positive microglia and CD68 punctate in the medial corpus callosum from mice administered with a control diet, 1 week cuprizone (1w), 4 week cuprizone (4w), and 4 week cuprizone + 1 week withdrawal (4 + 1w). (B) Number of CD68 punctate per microglia and (C) average puncta size. Maximum intensity projections (top panel) and orthogonal representations (bottom panel) of confocal z-stack images depicting (D) Iba-1 positive microglia and myelin basic protein (MBP) and (F) Iba-1 positive microglia and degraded myelin basic protein (dMBP). (E) Quantitative analysis of Iba-1 and MBP colocalization as a percent of total MBP+ area in the medial corpus callosum. (G) Volume of internalized dMBP per microglia in the medial corpus callosum. Data presented as mean±SEM and were analyzed using one-way ANOVA followed by Sidak’s post hoc test for multiple comparisons, n = 5-6, *p < 0.05, **p < 0.01, ***p < 0.001. Scale bar = 20 µm.

Previous studies have largely focused on the role of microglia in demyelination following weeks into cuprizone feeding or during remyelination upon cuprizone withdrawal [21–23]. Our findings here demonstrate that microglial activation occurs within a week of cuprizone feeding. We therefore sought to characterize the microglial functional state and the trigger of microglial activation in this early phase. Immunofluorescence labeling of CD68, a lysosomal protein associated with phagocytosis [24], showed a profound increase in CD68 expression with cuprizone feeding ([F(3,19) = 6.363, p = 0.0036]; Fig 3A,B). The CD68 signal overlapped with the Iba-1 positive cells, indicating that the phagocytic response is predominantly mounted by microglia. Specifically, the number of CD68 puncta per microglia showed a trend towards a significant increase in the 1 w group (p = 0.055; Fig 3B) and a significant increase in the 4 w group (p = 0.001; Fig 3B) compared with the Control, with a statistical trend towards a decrease in the 4 + 1 w group (p = 0.061; Fig. 3B). Likewise, the average puncta size was greater with cuprizone administration ([F(3,19) = 5.542, p = 0.0066]; Fig 3A,C). The average puncta size in both the 1 w group (p = 0.012; Fig 3C) and the 4 w group (p = 0.008; Fig 3C) were greater than in the Control. There was no difference in puncta size between the 4 + 1 w group and the Control group. Overall, the CD68 expression data indicate that the rapid microglial CD68 expression triggered by cuprizone tends to subside upon feeding withdrawal.

Microglial CD68 expression is associated with an activated state primarily marked by intracellular transport and lysosomal processing of debris and proteins [24,25]. Microglia were previously shown to play a critical role in the clearance of myelin debris during remyelination [5]. Moreover, inefficient clearance of myelin debris following cuprizone withdrawal is associated with impaired remyelination [5,6,12]. Yet, the temporal dynamics of microglial activation and the precise triggers of microglia activation are not well-understood. Given our finding of dysmorphic myelin as early as 1 week into cuprizone administration (Fig 1E), we assessed whether the increase in microglial CD68 expression was associated with microglial engulfment of myelin. Confocal z-stacks of Iba-1 and MBP immunofluorescence in the medial corpus callosum showed an increased colocalization of MBP in Iba-1 positive cells with cuprizone administration ([F(3,18) = 11.85, p = 0.0002]; Fig 3D,E). The percent MBP colocalization with Iba-1 was significant at 1 week (p = 0.0001, 1 w v. Control; Fig 3D,E) and at 4 weeks (p = 0.0006, 4 w v. Control; Fig 3D,E). Given the dysmorphic appearance of MBP (Fig 1E) and greater percent MBP-microglia colocalization in the cuprizone administration groups, we next assessed whether microglia engulf degraded myelin following cuprizone administration. We found an increase in the volume of degraded MBP (dMBP) internalized by microglia ([F(3,19) = 6.332, p = 0.0037]; Fig 3F,G). Compared with the Control group, microglial engulfment of dMBP was greater in all three groups (p = 0.0046 v. 1 w, p = 0.0416 v. 4 w, and p = 0.0035 v. 4 + 1 w; Fig 3F,G). Overall, these results indicate that cuprizone administration within a week triggered a microglial phagocytic response, characterized by engulfment of degraded myelin.

### One week of cuprizone administration increased phagocytosis-related gene expression while suppressing inflammatory cytokine response

**Fig 4:**
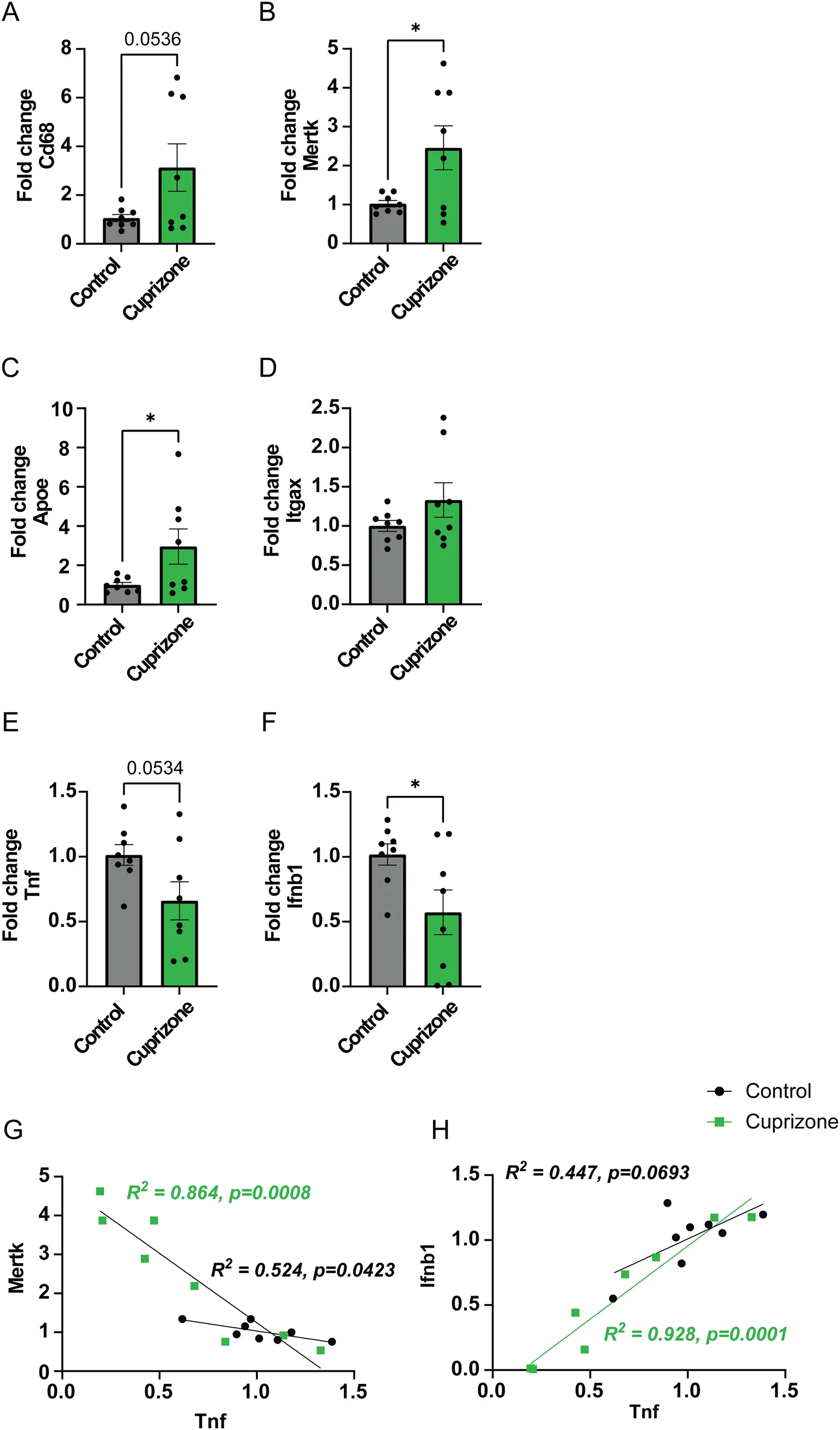
One week of cuprizone administration increased phagocytosis-related gene expression while suppressing inflammatory cytokine response. Real-time quantitative PCR analysis of Percoll-enriched cells from mice exposed to one week of cuprizone diet or control. mRNA expression of (A) Cd68, (B) Mertk, (C) Apoe, (D) Itgax, (E) Tnf, and (F) Ifnb1. Data presented as mean±SEM and were analyzed using a two-tailed unpaired t-test. n = 8, *p < 0.05. Scatterplots of the association between the gene expression changes: (G) Mertk and Tnf, (H) Ifnb1 and Tnf. Data analyzed using simple linear regression.

Microglia are dynamic surveyors of the brain microenvironment that rapidly respond to inflammatory insult and injury through diverse mechanisms, including cytokine and chemokine production, antigen presentation, and phagocytic response [26,27]. Therefore, we next characterized the gene expression changes associated with the microglial response during early demyelination. Specifically, we examined gene expression changes in Percoll-enriched cells isolated from the brain following a week of cuprizone administration (Fig 4). Real-time quantitative PCR showed a statistical trend towards an increase in *Cd68* mRNA levels in the Cuprizone group (p = 0.054; Fig 4A). The mRNA expression of *Mertk*, a tyrosine kinase receptor critical for clearance of apoptotic cells and myelin debris [6,17], was upregulated with cuprizone administration (p = 0.025; Fig 4B). Cuprizone also increased the expression of the gene, *Apoe*, which is critical in lipid metabolism and is upregulated in activated microglia [18] (p = 0.0484, Fig 4C). *Itgax*, a gene expressed in disease-associated microglia, was not significantly altered between the Cuprizone and Control groups [18] (Fig 4D). We next examined the expression of *Tnf* and *Ifnb1*, which are inflammatory mediator genes previously shown to be expressed in microglia following five weeks of cuprizone administration [10,13]. To examine whether these changes previously reported at five weeks are also present in the early demyelination phase, we measured *Tnf* and *Ifnb1* levels in our Percoll-enriched samples collected at one week of cuprizone feeding. Following one week of cuprizone administration, *Tnf* mRNA levels showed a trend towards a decrease (p = 0.053, Fig 4E), while *Ifnb1* was significantly decreased in the Cuprizone group compared with the Control (p = 0.035, Fig 4F). Both Control and Cuprizone groups showed a significant inverse correlation between *Mertk* expression and *Tnf* expression (p = 0.0423 in the Control group, p = 0.0008 in the Cuprizone group, Fig 4G). Higher *Mertk* expression was associated with lower *Tnf* expression in both Control and Cuprizone groups, and this relationship was significantly greater in the Cuprizone group than in the Control (significant difference between the slope of the Control line versus the Cuprizone line; p = 0.008; Fig 4G). In addition, *Tnf* expression was positively correlated with *Ifnb1* expression in the Cuprizone group, an effect not observed in the Control (p = 0.0693 in the Control group, p = 0.0001 in the Cuprizone group, Fig 4H). Overall, these findings demonstrate that the immediate response to cuprizone administration is marked by an augmented phagocytic state and a suppressed inflammatory response.

### Cuprizone enhanced inflammatory cytokine expression and suppressed phagocytosis *in vitro*

**Fig 5:**
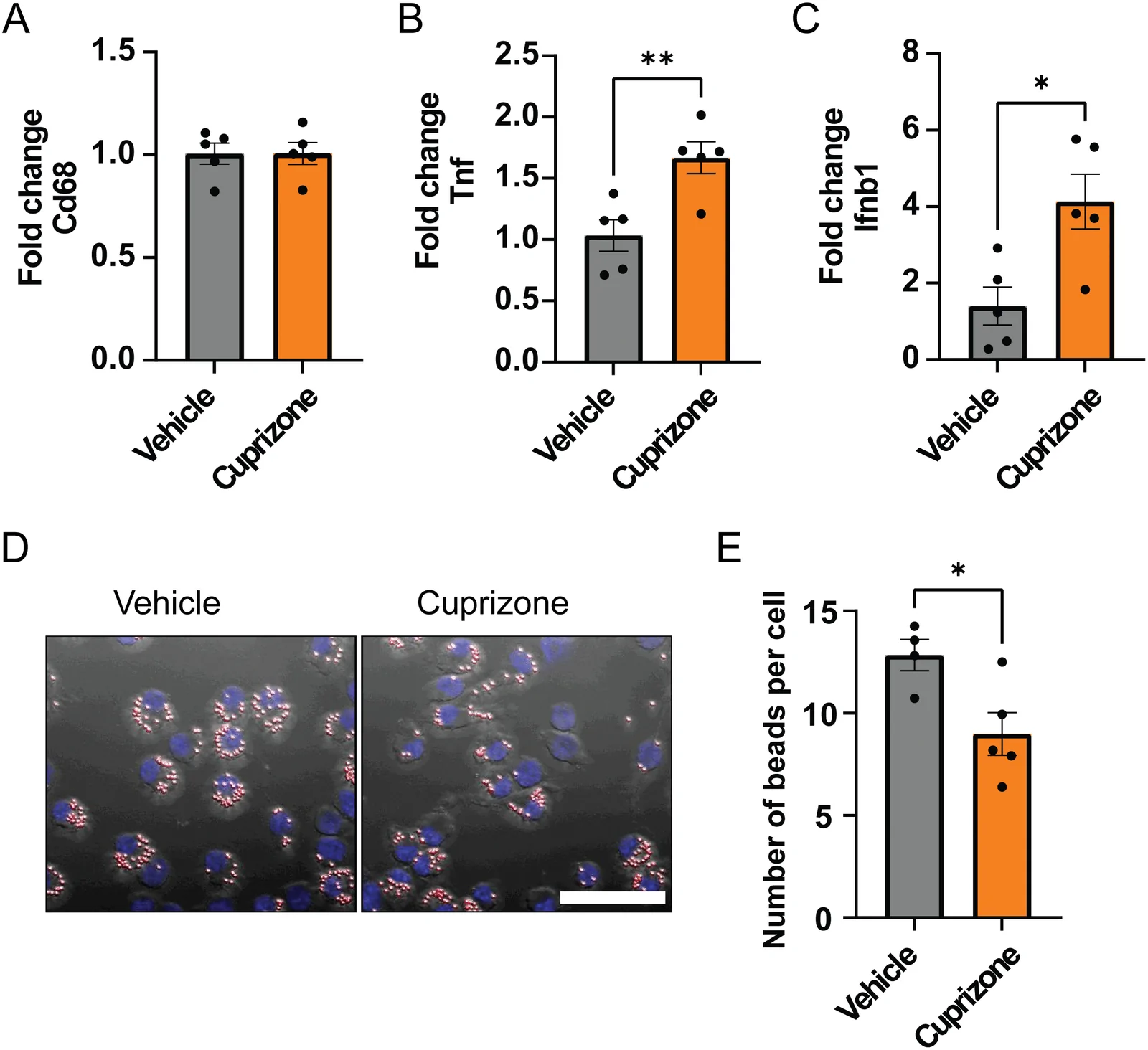
Cuprizone enhanced inflammatory cytokine expression and suppressed phagocytosis in vitro. Real-time quantitative PCR analysis of BV-2 cells following treatment with 25 µM cuprizone or vehicle for 24 hours. mRNA expression of (A) Cd68, (B) Tnf, and (C) Ifnb1. In a separate experiment, BV-2 cells were treated with fluorescence-labeled latex beads in presence of 25 µM cuprizone or vehicle for 24 hours. (D) Representative z-stack images of BV-2 cells with phycoerythrin-tagged beads (pink) and nuclei stained with DAPI (blue). (E) Quantification of beads per cell. Data are presented as mean±SEM and were analyzed using a two-tailed unpaired t-test, n = 5, *p < 0.05, **p < 0.01. Scale bar = 20 µm.

Although microglial activation in the cuprizone model of demyelination is well-documented, the potential effects of cuprizone directly on microglia remain unclear [3]. Here, we used an *in vitro* cell culture system to examine the effects of cuprizone on microglial BV-2 cells. Contrary to the findings *in vivo*, there was no difference in *Cd68* mRNA levels following 24 hours of cuprizone treatment (Fig 5A). mRNA levels of *Tnf* (p = 0.008, Fig 5B) and *Ifnb1* (p = 0.014, Fig 5C) were significantly greater in the Cuprizone group compared with the Control.

To assess whether cuprizone directly affects phagocytosis, we treated BV-2 cells with fluorescence-tagged latex beads in presence of cuprizone for 24 hours. The number of beads engulfed per cell was significantly lower in the Cuprizone condition compared with the Vehicle (p = 0.025, Fig 5D,E). Overall, these findings show that contrary to the findings *in vivo,* cuprizone suppressed phagocytosis *in vitro* in the BV-2 cells.

### Cuprizone administration *in vivo* reduced microglial CD68 expression independent of oligodendrocytes

**Fig 6:**
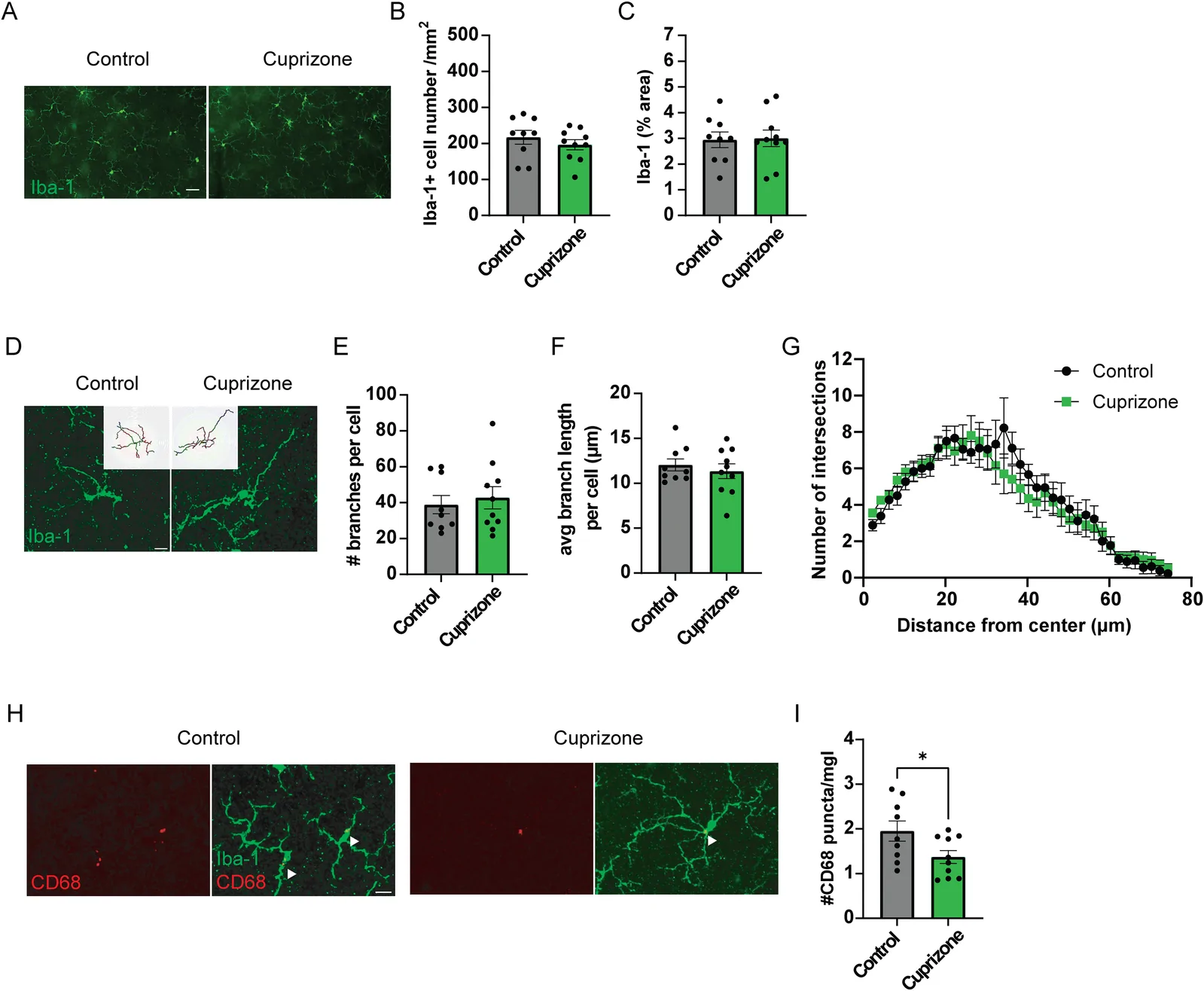
Cuprizone administration in vivo reduced microglial CD68 expression independent of oligodendrocytes. Mice were placed on a cuprizone diet for a week, and retina were collected for immunofluorescence labeling and analysis of microglia. (A) Representative images and quantifications of (B) Iba-1 positive cell number and (C) Iba-1% area in the retina. Scale bar = 50 µm. (D) Representative images depicting maximum projection intensity of Iba-1 positive microglia from confocal z-stacks. Skeletal analysis and quantification of (E) microglial branches and (F) average branch length per microglia. (D, inset) Representative reconstructions and (G) Sholl analysis quantification of Iba-1 positive microglia in the ganglion cell layer. (H) Representative confocal z-stack images of Iba-1 positive microglia and CD68 punctate in the ganglion cell layer of the retina following a week of cuprizone diet or control. (I) Quantification of CD68 puncta per microglia. Data presented as mean±SEM and were analyzed using a two-tailed unpaired t-test, n = 9-10, *p < 0.05. Scale bar = 20 µm.

Given that the microglial response to cuprizone administration in the corpus callosum was concurrent with oligodendrocyte death and demyelination, the extent to which cuprizone directly contributed to microglial activation cannot be determined. However, due to the absence of oligodendrocytes and myelin in the retina [28], the retinal microenvironment allows for an examination of the *in vivo* effects of cuprizone on microglia independent of oligodendrocyte death and myelin loss. We, therefore, assessed microglia profile in the ganglion cell layer of the retina following a week of cuprizone administration. Immunofluorescence labeling of Iba-1 cells showed no difference in microglia number (Fig 6A,B) or percent area (Fig 6C) with cuprizone administration. Sholl analysis and skeletal analysis revealed no significant difference in microglial morphology with cuprizone administration (Fig 6D-G). However, contrary to our findings in the corpus callosum, the retinal microglia in the Cuprizone group contained fewer CD68 puncta compared with the Control (p = 0.0405; Fig 6H,I). Overall, these findings reveal that cuprizone exerted direct effects on microglia *in vivo*, marked by reduced CD68 expression in absence of morphological remodeling.

## Discussion

The microglial response to cuprizone-induced demyelination has largely been studied weeks into cuprizone administration, when demyelination reaches peak levels [21,22]. The early triggers that drive microglia activation and the temporal shifts in microglial response over the course of demyelination remain unclear. Here, we report a robust microglial response – characterized by profound morphological remodeling and engulfment of dysmorphic myelin – in the corpus callosum within a week of cuprizone administration. This microglial phagocytic response was paralleled by suppression of inflammatory signaling processes. We further show that cuprizone exerted direct effects on microglia *in vitro* and *in vivo*. Notably, the direct response to cuprizone in microglia sharply contrasts the microglial response in the demyelinating microenvironment.

Microglial activation is a key feature in several chronic neurodegenerative conditions, including multiple sclerosis. The prolonged microglial response in chronic pathology involves a dynamic and complex response that has led to the characterization of microglia as “protectors turned destroyers” [11]. In order to track the trajectory of microglia response and functional shift, we examined microglia activation in the corpus callosum at various timepoints following cuprizone administration. Previous studies have reported an increase in microglia population in the corpus callosum between two and four weeks [10,29]. Here we show a significant expansion of microglia number and area in the medial corpus callosum within a week of cuprizone administration. This expansion was associated with a trend toward increased protein and mRNA expression of CD68, a lysosomal protein associated with phagocytosis and degradation of debris [30]. Although myelin basic protein (MBP) levels did not statistically decline until four weeks of cuprizone administration, the dysmorphic and clumpy appearance of MBP within the first week demonstrated early loss of myelin integrity. This early loss of myelin integrity was paralleled by microglial engulfment of degraded myelin basic protein (dMBP) at the early timepoint. Taken together, cuprizone triggered a profound microglial reorganization and phagocytic response to dysmorphic myelin within a week.

Single cell transcriptomic studies have identified a disease-associated microglia (DAM)-like signature in microglia following five weeks of cuprizone administration [10,13,18]. This profile is characterized by upregulation of genes associated with phagocytosis and lipid handling, including, Apoe, Lpl, and Spp1 [10,13]. Studies also report elevated inflammatory response associated with interferons, tumor necrosis factor, and cellular stress in microglia [10,13,31]. To examine whether the phagocytic response and the inflammatory cytokine response occur concurrently in the early phase of demyelination, we measured gene expression changes in microglia-enriched brain samples following a week of cuprizone administration. We observed increased mRNA levels of *Mertk* and *Apoe*, genes enriched in microglia and shown to be critical for myelin debris clearance and lipid metabolism, respectively [18]. The gene, *Itgax*, previously shown to be expressed in DAMs was not altered in the cuprizone condition, indicating that the DAM feature is not fully apparent during the early microglial response to demyelination [18]. Notably, cuprizone administration lowered *Tnf* and *Ifnb1* mRNA levels, indicating that the brain microenvironment response to cuprizone at the early timepoint involves suppression of inflammatory cytokine signaling. Furthermore, there was a strong inverse relationship between *Mertk* and *Tnf* expression with cuprizone administration at this early phase. It is worth noting that the microglia-enriched samples in our study do not exclusively include microglia. Nonetheless, *Cd68* mRNA levels showed a trend towards a statistically significant increase in the Cuprizone condition, and our immunofluorescence results did not reveal CD68 expression outside microglia. Therefore, we believe that the qPCR results predominantly represent the microglial gene expression profile.

Overall, the current study demonstrates an initial, adaptive response to demyelination marked by heightened phagocytosis and suppression of inflammatory cytokines. Specifically, mRNA levels of *Mertk* increased within a week, indicative of a phagocytic response in microglia. A previous study showed that Mertk deletion was associated with diminished microglial activation during demyelination and delayed remyelination following cuprizone withdrawal, demonstrating a role for microglial *Mertk* in myelin debris clearance [6]. In contrast to the elevated *Mertk* expression, we found reduced expression of *Tnf and Ifnb1*, indicating that the phagocytic response to demyelination early on was inversely correlated with a proinflammatory cytokine response. Consistent with our findings, Camenish et al. showed that *Mertk* knockdown was associated with an enhanced *Tnf* response and exacerbated response to endotoxemia [32]. Several studies have identified *Mertk* as an inhibitory regulator of cytokine signaling through mechanisms involving the suppressor of cytokine signaling (SOCS) [33,34]. Therefore, in our current study, suppression of *Tnf* and *Ifnb1* expression during peak demyelination is likely attributable to upregulation of *Mertk*.

These findings, together with studies examining cuprizone-induced demyelination at five weeks, indicate that the initial neuroinflammatory response, which is primarily characterized by phagocytosis, progresses into a complex state that subsequently also includes a proinflammatory cytokine response [10,13]. A better understanding of this shift is key to unraveling the mechanisms underlying microglial transition from an adaptive to a maladaptive state. A recent study using an Alzheimer’s Disease model showed that preventing lipid accumulation in microglia by deleting the *Fit2* gene enhanced phagocytosis while reducing overall proinflammatory cytokine expression in the brain [35]. Follow-up studies will be required to examine the relationship between lipid accumulation, cytokine response, and cellular stress in microglia over a prolonged demyelination period. A limitation of our current study is that the gene expression changes were examined in microglia-enriched brain samples, which precludes observations of cell-type-specific changes. Nonetheless, the findings reveal key gene expression changes in the brain microenvironment during early demyelination.

Although cuprizone is believed to selectively deplete oligodendrocytes and trigger subsequent demyelination, the direct effects of cuprizone on microglia have not been investigated. In primary cultures of rat brain cells, cuprizone was shown to selectively deplete oligodendrocytes [19,36]. Nonetheless, whether cuprizone exerts direct effects on microglia in non-lethal ways is unknown. Here, we examined microglia in the retina, a brain region devoid of oligodendrocytes and myelin. A week of cuprizone diet did not alter microglial morphology in the retina. Strikingly, retinal microglia showed a mild but significant downregulation of CD68 with cuprizone administration, in sharp contrast with the effects in the corpus callosum. *In vitro*, cuprizone treatment suppressed phagocytosis in BV-2 cells, while increasing *Tnf* and *Ifnb1* mRNA levels. The *in vitro* phagocytosis assay was conducted using fluorescent latex beads rather than myelin debris. Although the beads are not physiological substrates of phagocytes, they serve as experimental substrates to probe phagocytic response under *in vitro* experimental conditions [37]. Taken together, the microglial response in the corpus callosum following cuprizone administration is primarily attributable to changes in the microenvironment and are not due to the direct effects of cuprizone on microglia.

We show with the comparative studies in the retina that cuprizone itself does not trigger a profound microglial response in absence of oligodendrocytes or myelin. The retina is a central nervous system region colonized by microglia in the same manner and time window as the rest of the brain [38,39]. However, due to the absence of oligodendrocytes, the retina provides a unique environment to test whether microglia respond directly to cuprizone. It is important to note that even in areas with oligodendrocytes and myelin, microglia do not elicit a uniform, tissue-wide response to perturbations. For example, intracerebroventricular administration of colony stimulating factor-1 (CSF-1) triggered proliferation and morphological remodeling in white matter microglia but not in the gray matter microglia. Likewise, the colony stimulating factor-1 receptor ligand, interleukin-34, caused microglia proliferation in the gray matter but not in the white matter [40]. These findings underscore the complex and context-specific mechanisms underlying microglia activation following stimulation [41].

Previous studies have shown that cuprizone withdrawal is sufficient to promote oligodendrocyte growth and remyelination [6,13]. In line with these findings, the recovery group that received four weeks of cuprizone and a week of standard chow diet (4 + 1w) showed an overshoot in oligodendrocyte numbers, indicating that oligodendrocyte expansion occurred rapidly following cuprizone withdrawal. Although the microglia number in the recovery group did not differ from the 4w cuprizone group, the partial restoration of microglial morphology and CD68 expression in the recovery group puts these microglia in an intermediate activation state. Microglial engulfment of myelin did not differ between the recovery group and the 4w cuprizone group, indicating that microglial clearance of myelin debris continues following cuprizone withdrawal, as reported in previous studies [3,5]. Corresponding with the rapid oligodendrocyte repopulation, the number of BCAS-1 positive early myelinating oligodendrocytes significantly declined at the 4 + 1 w period, compared with the Control. These findings indicate that the BCAS-1 cells may contribute to the pool of mature oligodendrocytes during early remyelination. Notably, we observed a steady reduction in BCAS-1 cell numbers throughout the four weeks of cuprizone administration, although this decrease was not statistically significant. Fard et al. also reported a significant decrease in the BCAS-1 cell number with cuprizone administration [20]. Together, these findings indicate that the early oligodendrocyte cell population may rapidly mobilize to compensate for the oligodendrocyte loss following cuprizone administration, but the continued presence of cuprizone precludes the survival and maturation of the new oligodendrocytes [42].

In conclusion, our novel findings indicate that microglia undergo marked morphological remodeling and engulf disintegrated myelin within a week of cuprizone administration. In this early phase, the phagocytic response genes were augmented while the inflammatory signaling genes were suppressed. Lastly, we show that cuprizone exerts direct effects *in vivo* and *in vitro* on microglia independent of demyelination.
